# Correction to ‘Incidence and predictors of heart failure with improved ejection fraction category in a HFrEF patient population’

**DOI:** 10.1002/ehf2.15209

**Published:** 2025-01-21

Solymossi, B. , Muk, B. , Sepp, R. , Habon, T. , Borbély, A. , Heltai, K. , Majoros, Z. , Járai, Z. , Vágány, D. , Szatmári, Á. , Sziliczei, E. , Bánfi‐Bacsárdi, F. , and Nyolczas, N. (2024) Incidence and predictors of heart failure with improved ejection fraction category in a HFrEF patient population. ESC Heart Failure, 11: 783–794. 10.1002/ehf2.14619.38124459 PMC10966271

The following affiliation of the first and last authors was not included in the published version: Károly Rácz Doctoral School of Clinical Medicine, Semmelweis University, Budapest, Hungary

The author links and affiliations should have read:

Balázs Solymossi ^1,2^, Balázs Muk ^1^, Róbert Sepp ^3^, Tamás Habon ^4^, Attila Borbély ^5^, Krisztina Heltai ^6^, Zsuzsanna Majoros ^7^, Zoltán Járai ^8^, Dénes Vágány ^7^, Ákos Szatmári ^9^, Erzsébet Sziliczei ^10^, Fanni Bánfi‐Bacsárdi ^1^, Noémi Nyolczas ^1,2^

^1^Gottsegen National Cardiovascular Center, Budapest, Hungary

^2^Károly Rácz Doctoral School of Clinical Medicine, Semmelweis University, Budapest, Hungary

^3^Division of Non‐Invasive Cardiology, Department of Internal Medicine, University of Szeged, Szeged, Hungary

^4^Division of Cardiology, First Department of Medicine, Medical School, University of Pécs, Pécs, Hungary

^5^Division of Cardiology, Department of Cardiology, Faculty of Medicine, University of Debrecen, Debrecen, Hungary

^6^Heart and Vascular Center, Semmelweis University, Budapest, Hungary

^7^Department of Cardiology, Central Hospital of Northern Pest‐Military Hospital, Budapest, Hungary

^8^South‐Buda Center Hospital, St Imre University Teaching Hospital, Budapest, Hungary

^9^Cardiology Outpatient Clinic, Institute for Aviation Medicine, Military Fitness, and Medicine, Hungarian Defence Forces, Kecskemét, Hungary

^10^Fejér County Szent György University Teaching Hospital, Székesfehérvár, Hungary

We apologize for this error.

